# Significance of Simultaneous Splenic Artery Resection in Left-Sided Portal Hypertension After Pancreaticoduodenectomy with Combined Portal Vein Resection

**DOI:** 10.1007/s00268-017-3916-8

**Published:** 2017-03-03

**Authors:** Kazuyuki Gyoten, Shugo Mizuno, Motonori Nagata, Toru Ogura, Masanobu Usui, Shuji Isaji

**Affiliations:** 1 Department of Hepatobiliary Pancreatic and Transplant Surgery, Mie University School of Medicine, 2-174 Edobashi, Tsu, Mie 514-8507 Japan; 2 Department of Radiology, Mie University School of Medicine, 2-174 Edobashi, Tsu, Mie 514-8507 Japan; 3 Clinical Research Support Center, Mie University Hospital, 2-174 Edobashi, Tsu, Mie 514-8507 Japan

## Abstract

**Background:**

In pancreaticoduodenectomy (PD) with resection of portal vein (PV)/superior mesenteric vein (SMV) confluence, the splenic vein (SV) division may cause left-sided portal hypertension (LPH).

**Methods:**

The 88 pancreatic ductal adenocarcinoma patients who underwent PD with PV/SMV resection after chemoradiotherapy were classified into three groups: both SV and splenic artery (SA) were preserved in Group A (*n* = 16), SV was divided and SA was preserved in Group B (*n* = 58), and both SV and SA were divided in Group C (*n* = 14). We evaluated the influence of resection of SV and/or SA on LPH after PD with resection of PV/SMV confluence.

**Results:**

The incidence of postoperative varices in Groups A, B and C was 6.3, 67.2 and 38.5%, respectively (*p* < 0.001), and variceal bleeding occurred only in Group B (*n* = 4: 6.8%). In multivariate analysis, Group B was the only significant risk factor for the development of postoperative varices (Groups B vs. A: odds ratio = 39.6, *p* = 0.001, Groups C vs. A: odds ratio = 8.75, *p* = 0.066). The platelet count ratio at 6 months after operation comparing to preoperative value was 0.93, 0.73 and 1.09 in Groups A, B and C, respectively (Groups B vs. C: *p* < 0.05), and spleen volume ratio at 6 months was 1.00, 1.37 and 0.96 in Groups A, B and C, respectively (Groups B vs. A and C: *p* < 0.01 and *p* < 0.05).

**Conclusion:**

In PD with resection of PV–SMV confluence, the SV division causes LPH, but the concomitant division of SV and SA may attenuate it.

## Introduction

In carcinoma of the pancreatic head and body, the portal vein (PV) and/or superior mesenteric vein (SMV) is frequently involved because of its anatomical relationship. Although PV/SMV invasion was previously considered to be a contraindication for resection, some patients with PV/SMV invasion who undergo combined resection of these vessels achieve long-term survival equivalent to that in those without vascular invasion [1, 2]. As we previously reported the efficacy of chemoradiotherapy (CRT) followed by surgery for pancreatic ductal adenocarcinoma (PDAC) of the head for borderline resectable (BR) and locally unresectable (LUR) tumors, more than 80% of the patients required pancreaticoduodenectomy (PD) with concomitant PV/SMV resection [3]. Especially in PD with resection of PV–SMV confluence, we had resected splenic vein (SV) without reconstruction. After division of SV, congested venous flow of SV produces varicose routes and results in splenomegaly, which are defined as left-sided portal hypertension (LPH), causing variceal bleeding and thrombocytopenia by hypersplenism [3, 4]. Variceal bleeding after LPH is repeatable or massive in some patients, resulting in fatal hypovolemic shock. Hypersplenism causes pancytopenia, resulting in anemia, compromised status and easy bleeding. Such complications disturb quality of life and continuing chemotherapy. LPH is a critical problem after PD with resection of PV–SMV confluence because postoperative prognosis of PDAC patients is recently improving [5].

To avoid postoperative LPH, additional concomitant surgical procedures have been reported: SV–inferior mesenteric vein (IMV) or SV-PV/SMV anastomosis and construction of splenorenal shunt [6, 7]. However, SV re-anastomosis is not simple because the length of resected SV becomes longer due to tumor invasion, and furthermore, we have to separate SV from the pancreatic parenchyma. For the treatments of postoperative LPH, splenectomy or partial spleen embolization (PSE) has been employed [8, 9], although splenectomy has a long-term risk of overwhelming infection in 1–2% of patients [10].

For locally advanced PDAC invading splenic artery (SA), we developed a new surgical technique of PD with splenic artery resection (PD-SAR) and revealed the balance between operative radicality and postoperative pancreatic function [11]. In addition, PD-SAR also has a possibility to avoid LPH because the concomitant resection of SV and SA is considered to attenuate congestion of SV by decreasing splenic arterial inflow into spleen.

There have been a few studies on the pathophysiology of LPH after PV–SMV confluence resection without SV reconstruction for PDAC patients, and standard criteria for the diagnosis of LPH after PD have not been established. The aims of the present study were to clarify the incidence and clinical features of LPH after combined resection of PV–SMV confluence without SV reconstruction, and to evaluate the efficacy of concomitant SV and SA resection, the so-called PD-SAR, on the attenuation of LPH, by using our original simple criteria for LPH and by examining postoperative changes of platelet count and spleen volume.

## Patients and methods

### Patients

**Fig. 1:**
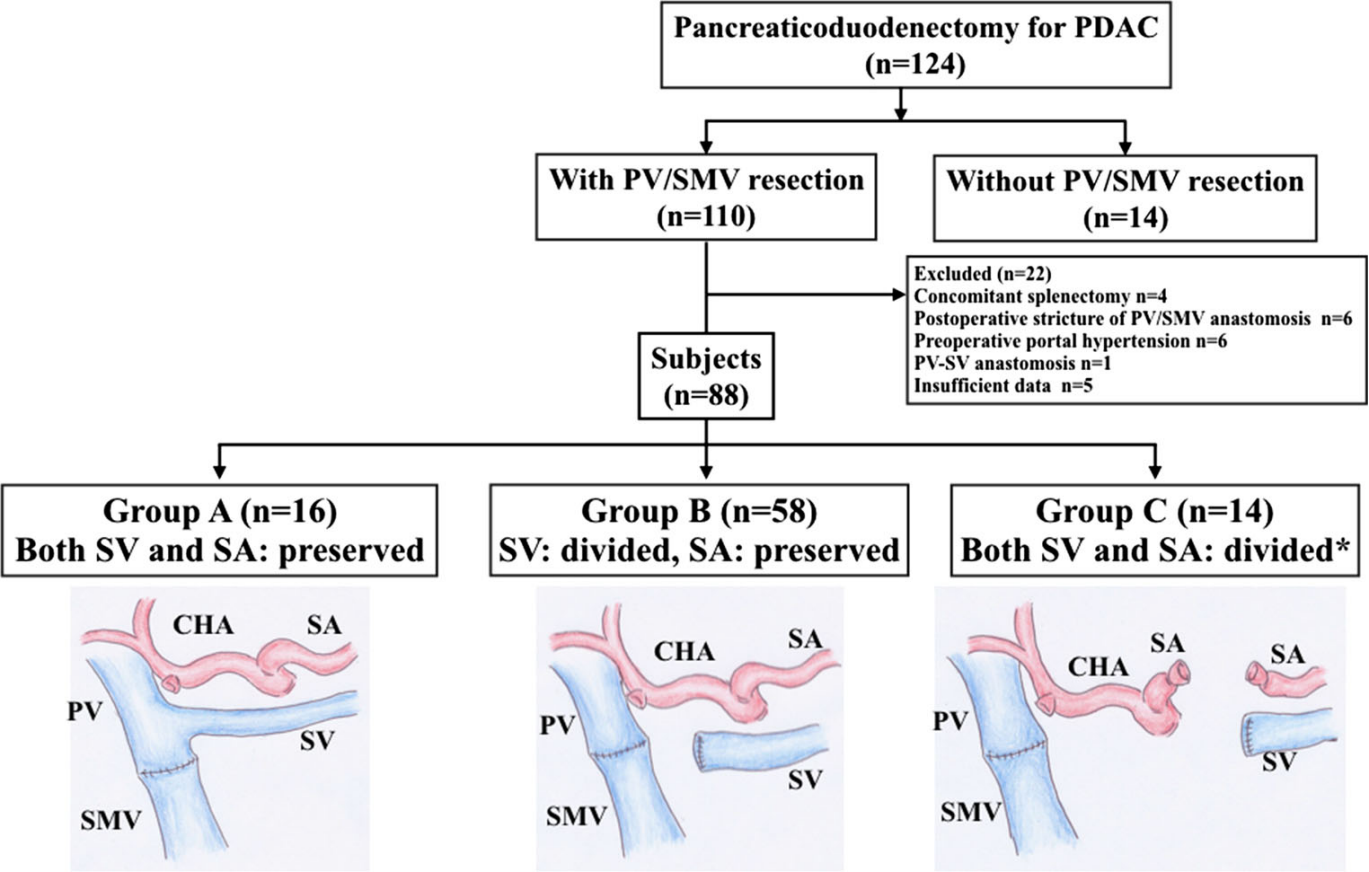
Classification of PDAC patients who underwent PD with PV/SMV resection. *PDAC* pancreatic ductal adenocarcinoma, *PV* portal vein, *SMV* superior mesenteric vein, *SV* splenic vein, *SA* splenic artery, *CHA* common hepatic artery. *PD-SAR: PD with splenic artery resection [11]

Between April 2005 and November 2015 at Mie University Hospital, we had enrolled 268 patients for our chemoradiotherapy (CRT) protocol reported previously [3], who were cytologically or histologically diagnosed as PDAC and having Union Internationale Contre le Cancer (UICC)-T3 and UICC-T4 tumors determined by using 64-slice multi-detector computed tomography (MDCT) [12]. Using our CRT database on the 268 patients, we retrospectively reviewed the electrical records of the 124 patients undergoing PD after CRT, in whom the 30-day postoperative and in-hospital mortality rates were 0 and 1.6% (*n* = 2), respectively. Among them, 110 (88.7%) underwent combined resection of PV/SMV resection (Fig. 1). The subjects of the present study were 88 patients by excluding 22 patients due to the following reasons: varices preoperatively existing due to PV and/or SV occlusion (*n* = 6), concomitant splenectomy (*n* = 4), intraoperative PV/SV anastomosis (*n* = 1), postoperative stricture of PV/SMV anastomosis (*n* = 6) and insufficient data (*n* = 5) including inadequate follow-up (*n* = 4) and in-hospital death (*n* = 1). These 88 patients were classified into the three groups: both SV and SA were preserved in Group A (*n* = 16), SV was divided and SA was preserved in Group B (*n* = 58), and both SV and SA were divided in Group C (*n* = 14) (PD-SAR). In Group B, IMV was preserved in 19 patients and ligated in 39 patients. Preoperative resectability of the tumor was classified into resectable (R), borderline resectable (BR) and unresectable (UR) according to the National Comprehensive Cancer Network (NCCN) guidelines (2010) [13], based on the findings of MDCT as previously reported [3].

### Preoperative treatments

Our treatment protocol for gemcitabine-based chemoradiotherapy (gem-CRTS) has been reported previously [12]. PDAC patients were treated with three-dimensional conformal radiotherapy using the 4-field box technique from directions that avoided exposure of the kidney to reduce an organ at risk. The clinical target volume was defined as the gross tumor volume plus a 5-mm margin in all directions based on the CT image. The total radiation dose delivered was 45–50.4 Gy in 25–28 fractions (5 fractions/week). Between February 2005 and October 2011, patients were administered an infusion of single-agent gemcitabine at a dosage of 800 mg/m^2^ on days 1, 8, 22 and 29. From November 2011, gemcitabine plus S-1 (orally active combination of tegafur, gimeracil and oteracil) combination therapy had been adopted, in which patients were administered gemcitabine at a dosage of 600 mg/m^2^ on days 1, 15 and 29, and S-1 at a dosage of 60 mg/m^2^ from day 1 to 21.

### Surgical techniques

Surgical procedures of PD with Child reconstruction for PDAC had been standardized for resection technique as anterior approach to the superior mesenteric artery (SMA) [11, 14]. The anastomosis between PV and SMV was performed by 6-0 non-absorbable running suture. When the SV or IMV was involved, they were divided and not reconstructed. When tumor involvement of SA was identified, PD-SAR was employed [11]. Pancreaticojejunostomy was performed using the Pair-Watch suturing technique according to our previous report [15].

### Preoperative characteristics, surgical outcomes and pathological findings

We compared various factors in the three groups, including preoperative characteristics such as gender, age, UICC-T factor, resectability according to NCCN guidelines 2010 [12], surgical outcomes such as intraoperative blood loss, operation time, degree of postoperative complications according to the Clavien–Dindo (C-D) classification [16], pancreatic fistula according to the International Study Group on Pancreatic Fistula [17] and duration of hospital stay (DHS). We also collected data on pathological examinations of the resected specimen such as PV invasion and surgical margin status (R0, R1 and R2).

### Postoperative treatments and follow-up

Adjuvant chemotherapy regimen starting at 6–8 weeks after PD consisted of gemcitabine at a dosage of 800 mg/m^2^ biweekly for at least 6 months between 2005 and 2013 [3], and thereafter, it was converted to chemotherapy of S1 at dosage of 60 mg/m^2^. Patients had a monthly follow-up by physical examination and blood test, and abdominal enhanced MDCT every 3 months. To assess the degree of hepatic steatosis after PD, CT values of the liver were evaluated by CT at 6 months after PD according to our previous report [5].

### Assessment of LPH and related outcomes

**Fig. 2:**
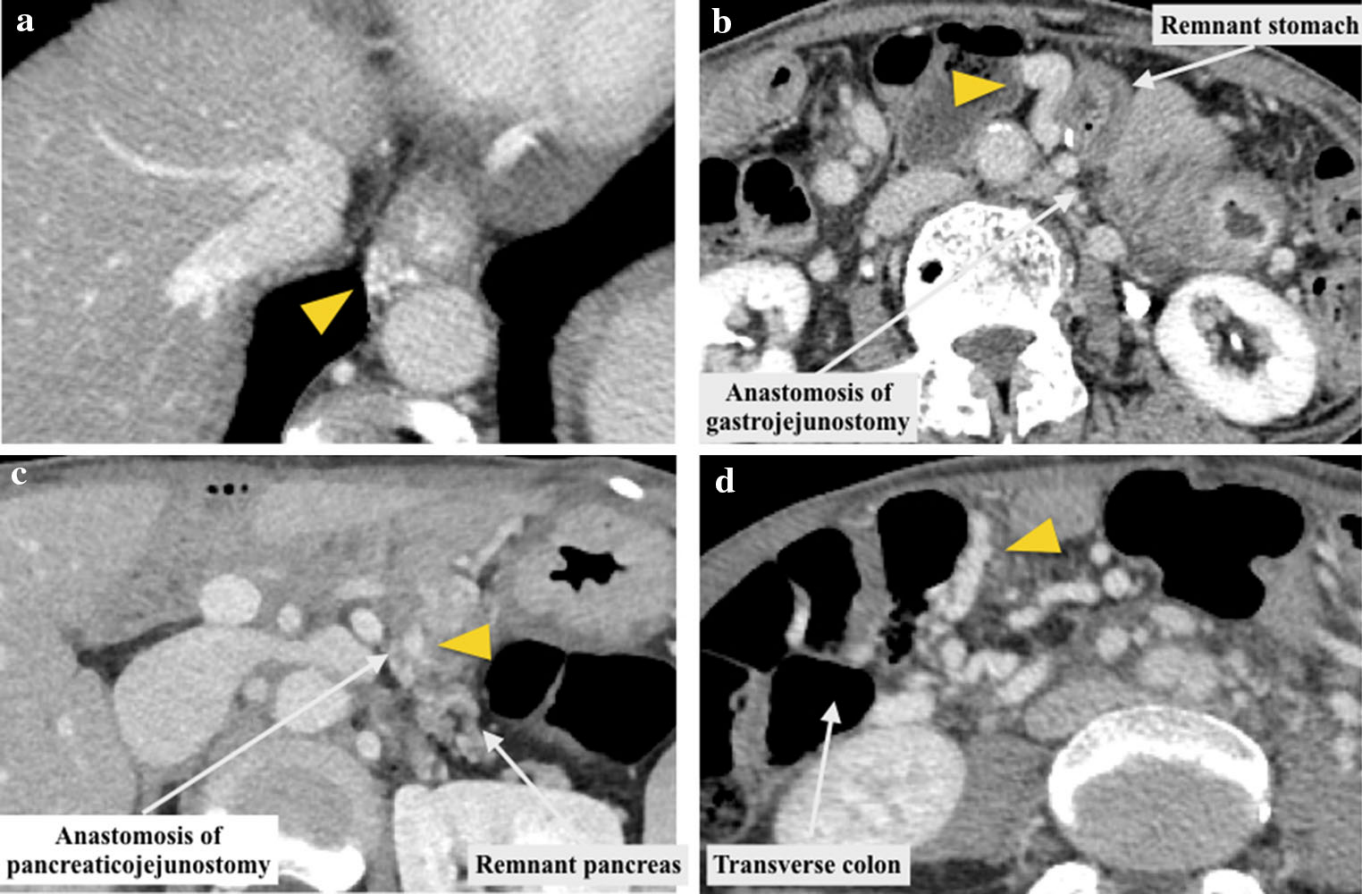
Types of varices developing after PD with PV/SMV resection. **a** Esophageal varices, **b** gastric varices, **c** pancreatic varices, **d** colonic varices. *Arrow head* indicates varices

To assess the development of LPH, the existence of intra-abdominal varices was evaluated at the time of 3–6 months postoperatively using enhanced MDCT by the radiologist (N. M) who was not informed of the patients’ characteristics and outcomes. Esophageal varices were diagnosed when the enhanced veins within the esophageal wall became apparently visualized as compared to the CT before operation (Fig. 2a). Gastric, pancreatic and colonic varices were diagnosed when the dilated veins, more than 5 mm in diameter, were detected within and/or around each organ (Fig. 2b–d).

Platelet count data were collected at preoperation and at 1, 3, 6 and 12 months after PD, respectively. Platelet counts ratio was calculated as the postoperative count divided by the preoperative count. The total spleen volume was estimated by tracing the spleen on each transverse CT image obtained at 2.0-mm intervals. Spleen volume was measured at preoperation and at 1, 3, 6 and 12 months after PD, respectively. Spleen volume ratio was calculated as the postoperative volume divided by the preoperative volume. We withdrew the evaluation of LPH when patients had massive liver metastasis, peritoneal dissemination, PV/SMV stenosis caused by tumor recurrence or succumbed.

All procedures and studies were carried out according to the ethical guidelines outlined by the Institutional Review Board (No. 1612).

### Statistical analyses

All continuous values were presented as mean with SD or range. Continuous variables were compared using one-way analysis of variance with Tukey’s multiple comparison test. If variables did not meet the homogeneity of variances assumption, the Games Howell post hoc test was used. Categorical variables were compared using Pearson’s Chi-squared test. The overall survival (OS) was calculated using the Kaplan–Meier method and tested using the log-rank test. Stepwise forward multiple logistic regression analysis of factors contributing to varices developing after operation was performed. Statistical data analysis was performed using the SPSS program, version 22.0 (SPSS, Chicago, Ill, USA). A *p* value less than 0.05 was considered statistically significant.

## Results

### Patients’ background and surgical outcomes

Patients’ background and surgical outcomes are shown in Table 1. In Group C, the rates of LUR and no achievement of surgical margin status R0 were significantly higher than those in the other two groups (*p* = 0.036 and *p* = 0.007, respectively). The development of varices was detected in 1 patient (6.3%) of Group A, in 39 (67.2%) of Group B and in 5 (35.7%) of Group C, showing significantly higher in Group B (*p* < 0.001). Variceal bleeding occurred in 4 patients (6.8%) only in Group B with no significance (*p* = 0.265).

**Table 1:** Patients’ backgrounds, surgical outcome and complications associated with LPH

	Group A (*n* = 16)	Group B (*n* = 58)	Group C (*n* = 14)	*p*
Age	67.2 (47–83)	65.5 (41–83)	69.5 (60–82)	0.249
Male:female	8:8	38:2	5:9	0.099
G-CRT:GS-CRT	7:10	27:31	8:6	0.556
Tumor size (mm)	31.5 (15–60)	30.6 (15–51)	35.3 (16–80)	0.359
R:BR:LUR	1:14:1	1:46:11	**0:7:7**	**0.036**
Operation time (min)	525 (345–818)	571 (351–810)	576 (363–842)	0.354
Blood loss (mL)	1178 (200–4930)	1613 (110–11,937)	2065 (300–8422)	0.373
C-D ≥ grade III	1 (6.3%)	16 (27.6%)	3 (21.4%)	0.195
DHS (days)	31.0 (14–60)	**48.8 (16–184)**	37.8 (22–60)	**0.018**
pPV positive	2 (12.5%)	14 (24.6%)	7 (46.6%)	0.058
R0	15 (93.8%)	54 (93.1%)	**9 (64.3%)**	**<0.001**
CT values of the liver at 6 months after Op (HU)	44.3 (−6.9 to 74.0)	42.1 (−9.35 to 71.5)	38.0 (−40.8 to 61.4)	0.785
Development of varices	1 (6.3%)	**39 (67.2%)**	5 (35.7%)	**<0.001**
Esophageal varices	0	**19**	3	**0.026**
Gastric varices	0	15	2	0.059
Pancreatic varices	0	**12**	0	**0.028**
Colonic varices	1	20	4	0.086
Development of shunt	0	**11 (18.9%)**	0	**0.039**
Splenorenal shunt	0	4	0	
Gastrorenal shunt	0	6	0	
The other type	0	1	0	
Variceal bleeding	0	4 (6.8%)	0	0.265

Bold values indicate statistically significant (*p* < 0.05)

*LPH* left-sided portal hypertension, *G* gemcitabine, *S* S-1, *CRT* chemoradiotherapy, *R* resectable, *BR* borderline resectable, *LUR* locally unresectable, *C*-*D* Clavien–Dindo classification, *DHS* duration of hospital stays, *pPV* pathological invasion of portal vein, *R*0 complete resection, no microscopic residual tumor

The early postoperative complications defined as C-D grade III or more were found in 1 patient (6.25%) of Group A, in 16 (27.5%) of Group B, and in 3 (21.4%) of Group C, showing no significant differences. In Table 2, types of early postoperative complications are shown. There was no surgical mortality case (C-D grade V); however, one patient in Group C who had severe diabetic mellitus and developed epidural abscess at postoperative day (POD) 39 died of lung metastasis and pleural dissemination at POD 136 during the hospital stay. In Group C, there were no complications related to SA resection such as splenic infarction, insufficiency of blood flow into pancreatic tail or remnant stomach, and aneurysm of the cut end of the SA.

**Table 2:** Early post-operative complications (Clavien–Dindo classification)

	Group A (*n* = 16)	Group B (*n* = 58)	Group C (*n* = 14)
Number of patients	1 (6.25%)	16 (27.5%)^a^	3 (21.4%)^a^
Grade IIIa	0	Intractable ascites [5] Pleural effusion [2] Pancreatic fistula [2] Biliary fistula [5] Anastomotic aneurysm of HA [3] Liver abscess [1]	Intractable ascites [1] Pleural effusion [1] Biliary fistula [1] Gastric ulcer [1]
Grade IIIb	Ileus [1]	Colonic anastomotic leakage [2]	Epidural abscess [1]
Grade IV	0	Sepsis [1]	0
Grade V	0	0	0

*PV* portal vein, *HA* hepatic artery

^a^Some patients experienced multiple postoperative complications

### LPH after PD with PV/SMV resection

**Fig. 3:**
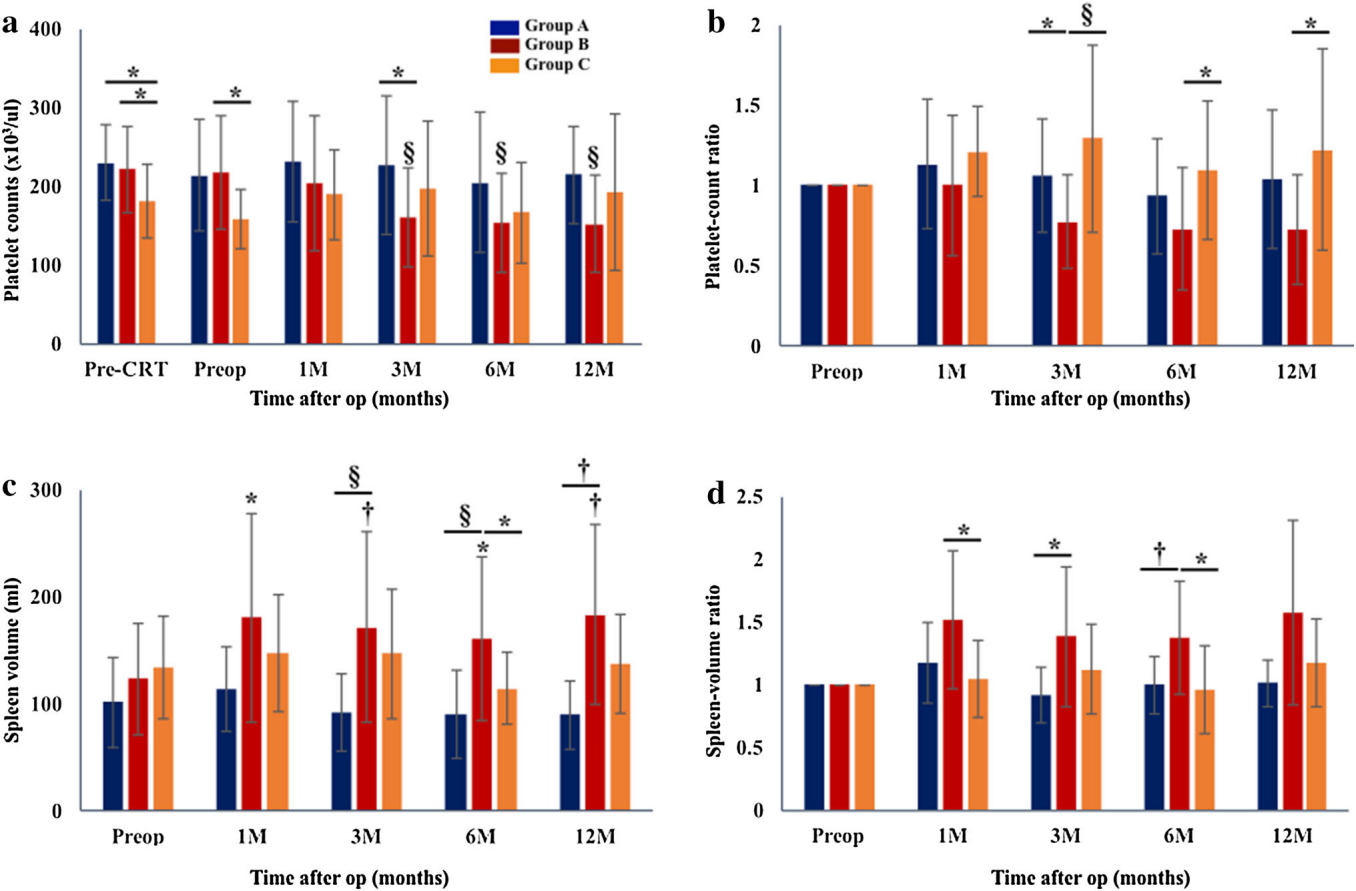
Pre- and postoperative changes in platelet counts (**a**), platelet count ratio (**b**), splenic volume (**c**), spleen volume ratio (**d**). Mean platelet count and the ratio in Group B began to decrease from 3 months after operation. In Groups A and C, the postoperative platelet count and ratio were comparable to preoperative values. Patients’ number in Group A/B/C was 16/58/14, 16/58/14, 16/58/14, 15/58/14, 13/42/11 and 8/29/7 at pre-CRT, Preop, 1, 3, 6 and 12 M, respectively. Mean spleen volume in Group B began to increase from 1 M after op, and was significantly higher at 3, 6 and 12 months than that in Group A. The ratios in Group B were significantly higher at 1 and 6 months than that in Group C. Patients’ number in Group A/B/C was 16/58/14, 8/45/11, 11/54/10, 14/41/12 and 8/28/7 at Preop, 1, 3, 6 and 12 M, respectively. *p < 0.05, ^†^ *p* < 0.01, ^‡^ *p* < 0.005, ^§^ *p* < 0.001. *CRT* chemoradiotherapy, *Preop* preoperation, *M* month

As shown in Fig. 3a, platelet counts in Group B were significantly decreased at 3, 6 and 12 months after operation compared to preoperative values (*p* < 0.01, *p* < 0.005, *p* < 0.001, respectively). In contrast, platelet counts in Groups A and C did not decrease significantly. There was a significant difference between Groups B and C before operation, and between Groups A and B at 3 months after operation. Preoperative platelet counts in Group C were significantly lower than those in Group B (203 × 10^3^/μL vs. 158 × 10^3^, *p* = 0.011). Platelet counts even before CRT were significantly lower than those in Groups A or B (180 × 10^3^/μL vs. 229 × 10^3^ or 221 × 10^3^, *p* = 0.036 or *p* = 0.003). As for platelet count ratio (Fig. 3b), the ratios in Group B were significantly lower at 3, 6 and 12 month compared to Group C (0.77 ± 0.29 vs. 1.29 ± 0.58, 0.73 ± 0.38 vs. 1.09 ± 0.43, 0.72 ± 0.34 vs. 1.22 ± 0.62, *p* < 0.05, respectively). The ratio in Group A at 6 months was 0.93 ± 0.36, showing no significant difference compared to that in Group B (0.73 ± 0.38, *p* = 0.236).

As shown in Fig. 3c, spleen volumes in Groups B were significantly higher at 3, 6 and 12 months compared to Group A (*p* < 0.001, *p* < 0.001 and *p* < 0.01, respectively), and also significantly higher at 6 month compared to Group C (*p* < 0.05). Additionally, spleen volumes in Group B were significantly increased from 1 month thereafter compared to preoperative values (*p* < 0.001 and *p* < 0.01, respectively). As for spleen volume ratio (Fig. 3d), the ratios in Group B were significantly higher at 1 and 6 months compared to Group C (1.52 ± 0.54 vs. 1.04 ± 0.31, 1.37 ± 0.45 vs. 0.96 ± 0.34, *p* < 0.05 and *p* < 0.05, respectively), and also significantly higher at 3 and 6 months compared to Group A (1.38 ± 0.56 vs. 0.91 ± 0.22, 1.37 ± 0.45 vs. 1.00 ± 0.22, *p* < 0.05 and *p* < 0.01, respectively). Between 19 patients with IMV preservation and 39 patients with IMV ligation in Group B, there were no differences in the incidence of variceal development, platelet count and ratio, and spleen volume and ratio at 6 months after PD (*p* = 0.312, *p* = 0.379 and *p* = 0.521).

Table 3 summarizes the characteristics of the four patients who developed variceal bleeding and required treatments. The first patient had hypovolemic shock due to massive bleeding from pancreaticojejunal varices at 6 months after PD, and underwent emergent splenectomy. On the next day after splenectomy, enhanced CT confirmed no more bleeding from pancreaticojejunal varices and shrinking of coexisting colic varices. The second patient had massive gastrointestinal bleeding at 6 months after PD: Firstly, emergency endoscopic clipping was tried but failed. Secondly, transarterial embolization was performed, but bleeding points could not be detected. Finally, distal gastrectomy and re-anastomosis of gastrojejunostomy by emergency laparotomy was tried, but patients died of disseminated intravascular coagulations. The third patients had hemorrhage from esophageal varices at 18 months after PD and required endoscopic variceal ligation twice. The fourth patient had rectal bleeding at 98 months after PD. Emergency enhanced CT revealed varices along the transverse colon and its mesenterium; 3D-CT demonstrated the dilated marginal veins along the transverse colon and the dilated vein communicating with the superior mesenteric vein. Colonoscopy revealed transverse colonic varices with hemorrhage which were treated by endoscopic injection sclerotherapy. Finally, the patient underwent transverse colectomy.

**Table 3:** Detail of four patients who developed varies bleeding

*N*	Age/sex	Variceal type	Months after op	Plt × 10^3^ (/μL) [Ratio]	SV (mL) [Ratio]	Treatments	Months after treatments
1	62/F	C, P	6	122 [0.56]	175 [1.24]	Splenectomy	9
2	74/F	G	6	127 [0.91]	156 [0.96]	Endoscopic clipping, TAE, gastrectomy	Death from DIC^a^
3	66/M	E	18	150 [0.66]	141 [3.47]	EVL	98
4	77/F	C	96	146 [0.69]	63.4 [1.04]	Colectomy	13

*M* male, *F* female, *E* esophageal, *G* gastric, *P* pancreatic, *C* colonic, *op* operation, *SV* spleen volume, *Plt* platelet counts, *TAE* transcatheter arterial embolization, *EVL* endoscopic variceal ligation, *DIC* disseminated intravascular coagulation

^a^Concomitant with jejunal ulcer

### Overall survival

**Fig. 4:**
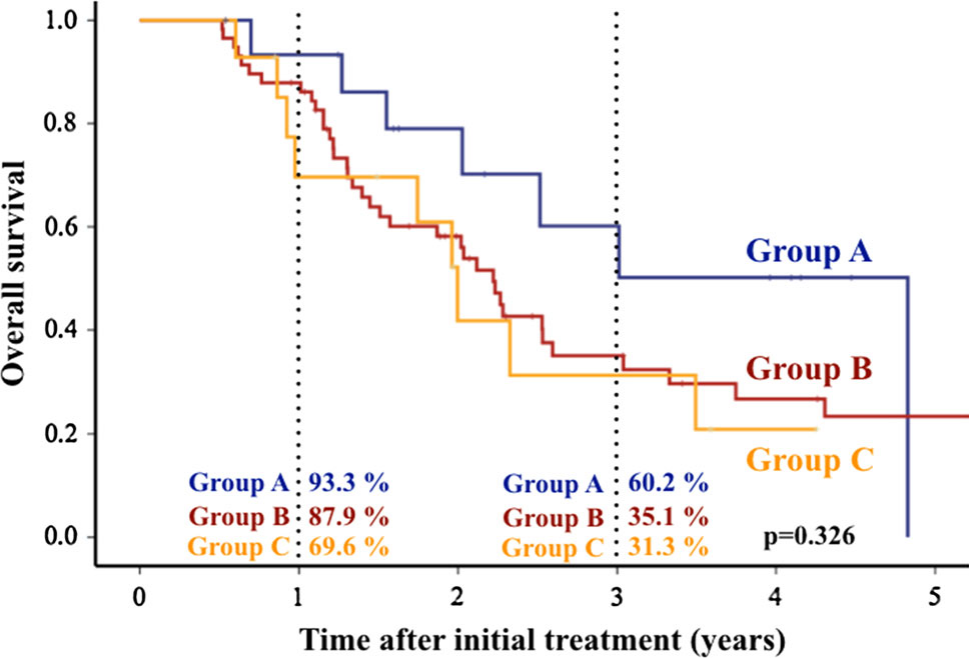
Overall survival after initial treatment. There were no significant differences in overall survival among three groups (*p* = 0.326)

Overall survival curves after initial treatment are shown in Fig. 4. One-year survival rate after initial treatment was 93.3%, 87.9 and 69.6 in Groups A, B and C, respectively. Three-year survival after initial treatment was 60.2%, 35.1 and 31.3 in Groups A, B and C, respectively. There were no significant differences in OS among the three groups (*p* = 0.326).

### Multivariate analysis of perioperative factors contributing to varices developing

In multivariate analysis of risk factors for varices developing, we evaluated the following factors: Groups A, B and C (SV or/and SA resection), age, gender, tumor size, preoperative platelet count, spleen volume, operation time, intraoperative blood loss, C-D classification ≥ grade III, pathological examinations of the resected specimen such as PV invasion and surgical margin status, DHS, CT values of the liver at 6 months after operation. As a result, Group B (SV was divided and SA was preserved) was the only significant risk factor for the development of postoperative varices (Table 4). Occurrence rate of varices developing in Group B was about 40 times as much as that in Group A (Odd’s ratio 39.6, *p* = 0.001). In addition, occurrence rate of varices developing in Group C was about 9 times as much as that in Group A (Odd’s ratio 8.75, *p* = 0.066).

**Table 4:** Multivariate analysis of factors contributing to the development of varices

	Odds ratio	95% confidence interval	*p*
Group A	1	–	–
Group B	39.6	4.70–334	0.001
Group C	8.75	0.863–88.6	0.066

## Discussion

LPH is a clinical syndrome due to outflow obstruction of SV, developing varices with hemorrhage and splenomegaly with thrombocytopenia. Outflow of SV is obstructed not only by pancreatic disorders, including acute and chronic pancreatitis, pancreatic pseudocysts and neoplasms [18–20], but by surgical procedures of SV division in PD with PV/SMV resection. The combined resection of PV/SMV is well accepted for pancreatic head cancer because it contributes to R0 achievement and is believed to have survival benefit without increasing postoperative morbidity and mortality [21, 22]. At the time of PV/SMV resection, SV is frequently divided for tumor invasion at PV/SMV confluence [23]; however, reconstruction of SV is controversial after anastomoses between PV and SMV reconstruction because there has been a lack of large data on the incidences and clinical features of LPH after resection of PV/SMV confluence based on the long-term outcomes.

Strasberg et al. [23] evaluated short outcomes of 10 PDAC patients who underwent combined resection of PV/SMV confluence without reconstruction of SV for the tumors of the neck of the pancreas involving the PV–SMV as well as the SV, being adjacent to SA. They concluded the SV division was safe and contributed to negative margin resection. Furthermore, they evaluated the development of varicose routes using CT or MRI in 5 PD cases in which the SV was divided approximately 2 cm to the left of its confluence with the SMV [24]. Six to eight months later, the longer length of residual SV provided opportunity for the development of alternate collateral pathway, which run along the omental arcade, around and through the colon or through the mesentery, and usually terminated in the SMV. As the result, splenomegaly and variceal bleeding did not develop. In no-operation cases like pancreatitis, on the other hand, no residual patent splenic vein was left because the obstruction of the SV was located near the pancreatic tail or had a diffuse thrombosis. In such situation, it is difficult to develop alternate collateral pathway except for classical venous pathway in which blood flow is from spleen into short gastric veins, then into perigastric and intragastric veins, and finally into coronary and portal veins [25]. As the result, splenomegaly developed and caused gastrointestinal bleeding. They concluded that the division of SV in PD did not result in a pattern of venous collateral development like the LPH caused by pancreatitis. Recently, Nagoya University Group [26] evaluated long-term outcomes of the 81 PDAC patients who underwent SV–SMV confluence resection without SV reconstruction compared with those of the 60 PDAC patients who underwent PV/SMV resection with preservation of SV, by examining appearance of collateral veins on the CT images until 2 years postoperatively. As a result, the platelet counts were significantly decreased after SV transection than after SV preservation at 6 months postoperatively, and spleen volume tended to be higher after SV transection than after SV preservation, although the difference did not reach statistical significance until 2 years postoperatively. On the other hand, the incidence of collateral veins was already significantly higher preoperatively in the SV transection than in SV preservation: about 7 versus 0%, and also significantly higher at 6 months: about 24 versus 9%. These variables become similar in long-term follow-up between the two groups, and therefore, they concluded that SV reconstruction might be unnecessary. In their study, however, they treated variceal rupture in a few case. They included the patients who had collateral veins preoperatively probably due to PV/SMV occlusion in the SV transection group. In contrast, our present study excluded these patients with varices preoperatively existing due to PV and/or SV occlusion. This difference might influence the long-term outcomes which were different between the two studies, although early result until 6 months postoperatively is very similar.

The incidence and venous hemodynamics of collateral vein after PV–SMV confluence resection without SV reconstruction still remain unclear because standard criteria for diagnosis of LPH after PD have not been established and precise evaluation of venous hemodynamics is very difficult. Recently, Ono et al. [4] precisely analyzed the routes of collateral veins from the spleen in the 43 PDAC patients who underwent PV–SMV confluence resection without SV reconstruction by using dynamic MDCT studies in which veins emanating from the spleen were traced carefully on serial transaxial or reconstructed three-dimensional image. As a result, they found two predominant venous flow patterns from the spleen: One was varicose route in 27 patients (62.8%) of whom the flow from the spleen passed to colonic varices and/or other varicose veins and the other was non-varicose route in 16 patients of whom the flow passed through the splenocolonic collateral draining to PV or IMV. The patients with varicose route showed significantly greater splenic hypertrophy than those with non-varicose route: median spleen volume ratio 1.52 versus 0.94 (*p* < 0.001) at 4–8 months. Since none of the patients with the varicose route had the superior right colic vein (SRCV) preserved, they emphasized the importance of preservation of SRCV to avoid the development of varices. In our present study, incidence of varicose vein in Group B was 67.2%, which was similar to their study although the criteria were different. The SV division was a significant risk factor for postoperative varices developing.

To prevent postoperative LPH after PV–SMV confluence resection, the preservation of IMV branched from SV or anastomosis between IMV and SV had been reported in small number of patients with very short follow-up [5, 7], under the hypothesis that it reduces congestion of the stomach and spleen. To evaluate venous hemodynamics between IMV and SV, Misuta et al. [27] performed venography through the celiac angiography at early postoperative day in the 12 patients who had PV–SMV confluence resection and had preservation of SV–IMV confluence or reconstructive anastomosis between the SV and the IMV: 9 showed downward flow (from the SV to the IMV) and 3 showed upward flow (from the IMV to the SV). Postoperative CT scans demonstrated venous dilatation and splenomegaly in the upward flow group, whereas there were no venous dilation and splenomegaly in the downward flow group. Therefore, preservation of IMV branched from SV or anastomosis between IMV and SV does not always reduce congestion of the stomach and spleen. Ono et al. [4] also evaluated the impact of preservation of SV–IMV confluence on the development of LPH after PV–SMV confluence resection without SV reconstruction by comparing 27 patients with IMV resection and 16 without IMV resection. As a result, there was no significant difference in splenic hypertrophy ratio between patients with and without IMV ligation. Our present study revealed that there were no differences in incidence of variceal development, platelet count and ratio, and spleen volume and ratio regardless of IMV preservation.

To the best of our knowledge, there have been no previous studies on the impact of concomitant SV and SA resection (PD-SAR) on the development of LPH after PV–SMV confluence resection without SV reconstruction. PD-SAR is a simple procedure comparing to the techniques such as SV–IMV anastomosis and SV-PV/SMV anastomosis, and we had already proven that PD-SAR can maintain blood supply of the remnant pancreatic tail and spleen as well as postoperative pancreatic functions [11]. In fact, there were no surgical complications related to SA resection in the present study, revealing that PD-SAR prevented splenomegaly and maintained platelet counts. In patients who underwent PD-SAR (Group C), OS was comparable to those of the other two groups in spite of the fact that the rates of LUR and R1 were significantly higher.

Actually, we had developed PD-SAR procedure without an attempt to prevent from LPH after PV–SMV confluence resection; unexpectedly, however, this procedure was revealed to attenuate LPH. From these results, we recently performed the SA ligation using a clip when the PV–SMV confluence was resected. Consequently, the SA ligation or division might be a useful and simple procedure to prevent from LPH when resection of the PV–SMV confluence is performed.

In conclusion, the SV division is a risk factor for LPH, resulting in variceal developing, the decrease of platelet counts and the increase of spleen volume in PD with the PV/SMV resection. Concomitant SA division may attenuate the risk.
